# The Role of Selenoprotein Tissue Homeostasis in MetS Programming: Energy Balance and Cardiometabolic Implications

**DOI:** 10.3390/antiox11020394

**Published:** 2022-02-15

**Authors:** María Luisa Ojeda, Olimpia Carreras, Fátima Nogales

**Affiliations:** Department of Physiology, Faculty of Pharmacy, Seville University, 41012 Seville, Spain; ojedamuri11@us.es (M.L.O.); fnogales@us.es (F.N.)

**Keywords:** selenium, selenoprotein, metabolic syndrome, fetal programming, cardiovascular disease

## Abstract

Selenium (Se) is an essential trace element mainly known for its antioxidant, anti-inflammatory, and anti-apoptotic properties, as it is part of the catalytic center of 25 different selenoproteins. Some of them are related to insulin resistance (IR) and metabolic syndrome (MetS) generation, modulating reactive oxygen species (ROS), and the energetic sensor AMP-activated protein kinase (AMPK); they can also regulate the nuclear transcription factor kappa-B (NF-kB), leading to changes in inflammation production. Selenoproteins are also necessary for the correct synthesis of insulin and thyroid hormones. They are also involved in endocrine central regulation of appetite and energy homeostasis, affecting growth and development. MetS, a complex metabolic disorder, can appear during gestation and lactation in mothers, leading to energetic and metabolic changes in their offspring that, according to the metabolic programming theory, will produce cardiovascular and metabolic diseases later in life. However, there is a gap concerning Se tissue levels and selenoproteins’ implications in MetS generation, which is even greater during MetS programming. This narrative review also provides an overview of the existing evidence, based on experimental research from our laboratory, which strengthens the fact that maternal MetS leads to changes in Se tissue deposits and antioxidant selenoproteins’ expression in their offspring. These changes contribute to alterations in tissues’ oxidative damage, inflammation, energy balance, and tissue function, mainly in the heart. Se imbalance also could modulate appetite and endocrine energy balance, affecting pups’ growth and development. MetS pups present a profile similar to that of diabetes type 1, which also appeared when dams were exposed to low-Se dietary supply. Maternal Se supplementation should be taken into account if, during gestation and/or lactation periods, there are suspicions of endocrine energy imbalance in the offspring, such as MetS. It could be an interesting therapy to induce heart reprogramming. However, more studies are necessary.

## 1. Selenium and Metabolism Disorders

### 1.1. Selenium 

Selenium (Se) (Se ^34^ _79_) is an essential trace element mainly known for its antioxidant [1], anti-inflammatory, and anti-apoptotic properties as it is part of the catalytic center of different selenoproteins [2,3,4]. Adequate Se intake is essential for immune, endocrine, cardiovascular, reproductive, and nervous systems functions [5,6,7,8,9]. However, the margin between Se toxicity and its deficiency is very narrow [10]. Its role in the human body has been studied, especially for thyroid function, type 2 diabetes mellitus (T2DM), hypertension, obesity, inflammation, reproductive system, cancer, and cardiovascular disease. Se excessive and deficient dietary intake is associated with damaging health effects that have been characterized by a U-shaped relationship [11]. For this, a balanced intake of Se is crucial to maximizing the health benefits of selenium [12]. Recommendations for Se intake are: in adults 55–70 µg/day, in infants 15 µg/day, in children 20–30 µg/day, in pregnant women 65 µg/day, and in lactating mothers 75 µg/day [13], the tolerable upper intake level is limited to 300 µg/day [14]. 

Dietary Se is absorbed by the gastrointestinal tract (GIT) in its inorganic and organic forms. Organic forms of Se such as selenomethionine (SeMet) and selenocysteine (Sec) are absorbed in the small intestine through the same active sodium-dependent transport system as the amino acid methionine. Additionally, Sec may be absorbed using the same mechanism as cysteine [15,16,17,18]. Inorganic forms are absorbed by the same sodium-facilitated and energy-dependent systems as sulfate [19]. Moreover, selenate and selenite can be uptaken by non-mediated passive diffusion, with a slower absorption rate than the Se-organic compounds [20]. After intestinal absorption, Se forms enter the bloodstream and are predominantly taken up into the liver from the portal vein [21]; in this tissue they will be further metabolized, turning these inorganic forms into more bioavailable organic forms. So, in the human body, two metabolic pools of Se are predominant. One pool includes all forms of Se derived from inorganic selenite/selenide, including excretory Se metabolites and other intermediate products of selenite metabolism [22]. The second pool consists of organic forms of Se and SeMet-containing proteins. The extracellular Se forms, from inorganic and organic pools, are captured by the liver and other tissues such as muscle and mammary glands [23]. Later inside the cells, Sec, selenite, and selenide compounds form an intracellular metabolic reserve, whereas SeMet is incorporated into proteins in place of methionine. This amino acid is also converted to Sec via the transsulfuration pathway, and this Sec is transformed to selenide with the help of Sec-lyase enzyme. In the liver, inorganic forms are reduced to selenide by thioredoxin reductases or the glutathione (GSH) pathway. Then selenide is transformed to selenophosphate by the enzyme selenophosphate synthetase 2. Selenophosphate is used for selenoprotein synthesis. 

The selenoprotein synthesis (translational decoding process) begins when selenophospate reactions with phosphoseryl-tRNA yield Sec-tRNA[Ser]Sec. Sec amino acids are incorporated into polypeptide chains utilizing the UGA codon. Selenocysteine insertion sequence binding protein 2 (SBP2) binds to selenocysteine insertion sequence (SECIS) element which is located in the 3′-untranslated region (3′UTR) of selenoprotein mRNA and mediates the transfer of Sec-tRNA[Ser]Sec to the A-site of the ribosome which recognizes the UGA codon as the Sec integration codon [24]. The amino acid Sec can appear in the N or C-terminal part of protein according to two different groups of selenoproteins, representing the most important part of the active center of selenoproteins. Selenoprotein P (SelP) is the main selenoprotein produced in the liver, containing 10 Sec residues; so, it functions as a Se-transport protein to deliver Se to other tissues [25]. The different tissues need specific receptors to uptake SelP. Then, in the tissues, Se is used to synthetize new other selenoproteins [26,27,28]. The placenta, brain, and testes uptake SelP through receptor-mediated endocytosis using the low-density lipoprotein receptor-related protein 8 (LRP8), also known as ApoER2 [29,30]. Other tissues, like the kidneys, also use another membrane receptor, the megalin or LRP2. Se is differentially distributed in the body, following a tissue hierarchy that is even extended to intracellular mechanisms that prioritize the synthesis of specific selenoproteins [31,32,33]. This fact makes the studies that address the tissue distribution of Se and selenoproteins extremely complex.

Twenty-five selenoproteins are recognized in humans, with different biological functions (Table 1) [34,35], such as iodothyronine deiodinases (DIOs: 1, 2, and 3 families), responsible for thyroid function; or thioredoxin reductases (TXNRD1, 2, and 3), SelW, SelH, SelT, and SelV, involved in redox regulation processes [36]. However, most of them have antioxidant properties, like the glutathione peroxidases family (GPxs: GPx1–GPx8), which eliminate the excess of H_2_O_2_, and selenoprotein P (SelP), the main serum Se transporter [2]. Both of them are also related to the endocrine system and intracellular signaling, appetite, growth, and energy homeostasis [37,38]. 

Among all these selenoproteins, the TXNRD, GPx, and DIO families are the three best characterized [40]. They have different enzymes activities, although all of them require reductants to provide the electrons to make their catalytic redox cycle run. Thus, TXNRD-dependent reduction requires electron transfer from NADPH, FAD via, to Sec at their active site and finally to the substrate thioredoxin. In the GPx family, the catalytic redox cycle involves the oxidation of Sec to selenic acid by hydrogen peroxide and organic hydroperoxides, and reduction to the selenolate anion form by the GSH system [41,42]. Finally, the action mechanism of DIO selenoproteins involves the generation of an oxidized intermediate, which will be reduced by thiol-containing reductants and release iodide. 

In general terms, selenoproteins and Se can exert their main functions through different mechanisms. (1) By ROS-mediated stimulation of intracellular protein kinases in the cytoplasm and the nucleus, such as the mitogen-activated (MAP) kinase, the p38 kinase, and the c-jun/stress-activated kinase, all of them are involved in the growth responses of cells to stressful and inflammatory stimuli [43]. In this context, several studies have found that selenate is a stimulator of the tyrosine kinases, as happens in the insulin signaling cascade having insulin-mimetic effects [44,45,46], by contrast, high doses of selenite has shown to impair/dampen insulin signaling [47,48]. Therefore, Se compounds and selenoproteins play an important role in fuel metabolism processes [49,50]. (2) Another mechanism involves ROS-mediated covalent modification of thiol, cysteine, and tyrosine groups of proteins. (3) Additionally, Se can produce alterations in cellular redox state causing activation of transcription factors, such as NF-kB, Ap-1, and the glucorticoid receptor, leading to de novo gene expression. Thus, Se can affect transcription factors activation by either affecting DNA-binding strength or changing activation of the transcription factor by modulation of regulatory subunits, e.g., by phosphorylation. (4) Se regulates the expression of cell surface and nuclear receptors leading to alteration in cell growth, responsiveness, and behavior. (5) Finally, this element can regulate the cell death/survival signals.

**Figure 1:**
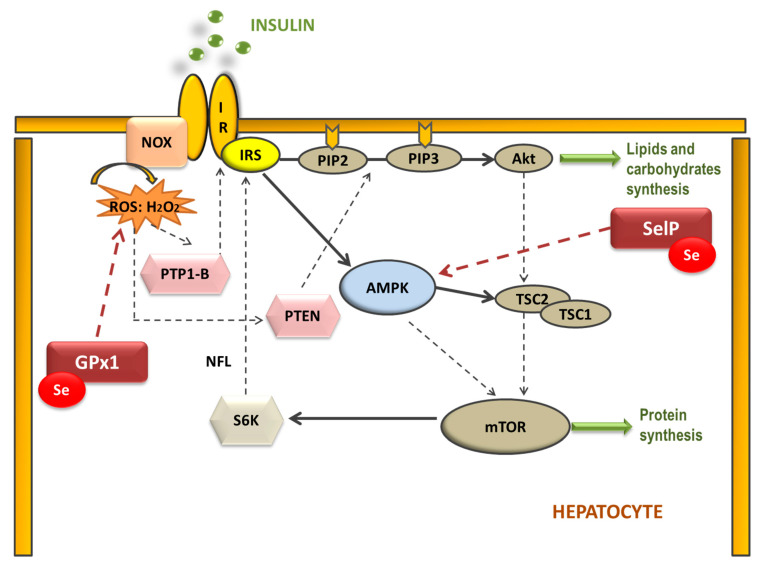
Selenoproteins’ possible implication in the hepatic insulin signaling pathway. The binding of insulin to the insulin receptor (IR) triggers consecutive phosphorylation (P) of downstream signaling molecules, resulting in activation of Akt. Additionally, NADPH oxidase (NOX)-mediated ROS production is stimulated by insulin. Reduction of H_2_O_2_ by GPx1 may attenuate insulin signaling, as H_2_O_2_ is required to inactivate the insulin counter-regulatory phosphatases PTP-1B and PTEN. Moreover, SelP could inhibit adenosine monophosphate-activated protein kinase (AMPK), a metabolic energy sensor, which negatively regulates protein synthesis through the inhibition of the mammalian target of rapamycin (mTOR)-S6 kinase (S6K) pathway, which also modulates IRS phosphorylation. (IRS: insulin receptor substrate; PIP2: phosphatidylinositol 4,5-bisphosphate; PIP3: phosphatidylinositol 3,4,5-trisphosphate; TSC1 and 2: tuberous sclerosis complex 1 and 2).

Therefore, selenoproteins have an important role in insulin resistance (IR) and metabolic syndrome (MetS) generation, modulating the reactive oxygen species (ROS) implicated in insulin signaling and the energetic sensor AMP-activated protein kinase (AMPK) [46,51]. Elevated dietary Se intake is associated with IR by increasing hepatic GPx1 activity, which decreases H_2_O_2_ levels (Figure 1). In this case, H_2_O_2_ acts as a second messenger formed after the activation of NADPH oxidases (NOX) when insulin binds to its receptor (IR). H_2_O_2_ is required to inactivate various insulin-signaling inhibitors as the protein tyrosine phosphatase 1B (PTP-1B) or the dual-specificity phosphatase PTEN by oxidation of essential SH groups [52]. PTP-1B inhibits the phosphorylation (activation) of insulin receptor substrate (IRS-1), while PTEN dephosphorylates phosphatidylinositol 3,4,5-trisphosphate (PIP3) at position 3′ to PIP2, depressing protein kinase B (Akt) signaling and triggering IR [53]. Thus, high Se levels augment hepatic GPx1 activity, which reduces H_2_O_2,_ decreasing the oxidative inhibition of PTP1B and PTEN and suppressing insulin signaling [54]. On the other hand, when SelP is increased in the liver it inhibits AMPK activity impairing insulin signaling transduction, and is recognized as a hepatokine that contributes to the beginning of hyperglycemia and IR [51]. Additionally, other mechanisms have been proposed to explain the role of SelP in IR using primary hepatocytes, describing that purified SelP induces a reduction in insulin-stimulated phosphorylation of IR, IRS-1, and Akt [55]. In addition, it is important to take in mind that supranutritional Se modifies the expression of transcriptional factors and enzymes related to carbohydrate, lipids, and protein metabolism in a tissue-selective manner, such as liver, skeletal muscle, and adipose tissue [50]. 

However, Se deficiency is also associated with IR [56,57]. Seale et al. found that when cellular Se recycling mechanisms are lacking, a decrease in GPx1 and SelP expression appears, which is related to an inhibition of insulin signaling; probably, among others, by leading to an excessive increase of H_2_O_2_ and oxidative stress (OS) [58]. These results have been recently confirmed by different authors, who concluded that a disruption on the selenocysteine lyase which mediates the Se recycling pathway, leads to hyper-adiposity, obesity, IR, and deep changes in metabolic homeostasis [59,60,61]. For these reasons, an appropriate Se homeostasis, neither high nor low, is important to maintain a correct oxidative balance in order to avoid metabolic disruptions. 

**Figure 2:**
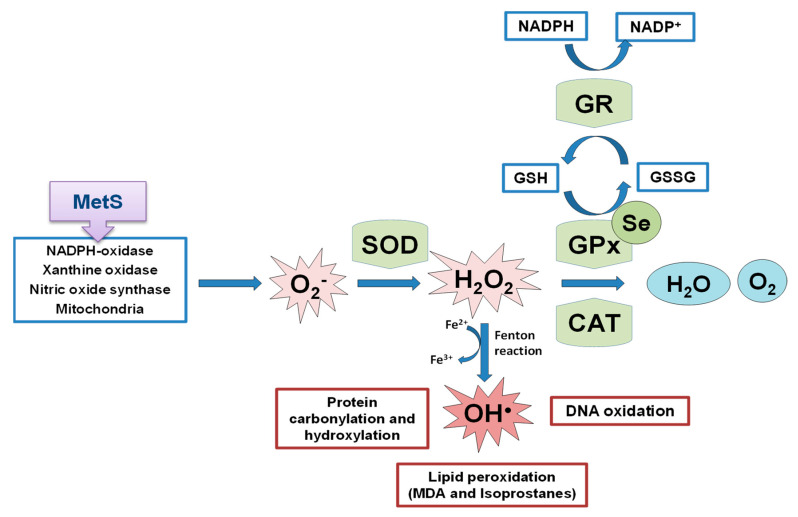
Schematic representation of endogenous antioxidant enzyme systems, the effect of the selenoprotein GPxs against the formed ROS. MetS implication in ROS generation. SOD: superoxide dismutase; GPx: glutathione peroxidase; CAT: catalase; GR: glutathione reductase; GSH: reduced glutathione; GSSG: oxidized glutathione; MDA: malondialdehyde.

OS is a state of negative imbalance between the excess of pro-oxidative compounds and the insufficient decomposition of those compounds by the antioxidant systems [62]. ROS in basal conditions are essential for cellular functions (such as in the insulin signaling cascade), whereas excessive levels of ROS cause damage to cells by the oxidation of lipids, DNA, and proteins [63,64,65], leading to cellular dysfunction including loss of energy metabolism, altered cell signaling and cell cycle control, genetic mutations, altered cellular transport mechanisms, and overall decreased biological activity, immune activation, and inflammation [66]. ROS include oxygen free radicals, such as the superoxide anion (O_2_^−^), the hydroxyl radical (^•^OH), and non-radicals, such as hydrogen peroxide (H_2_O_2_). They can also react with NO leading to peroxynitrite, the so-called RNS. The defense mechanism responsible for ROS inactivation included endogenous antioxidants, classified into enzymatic antioxidants (GPxs, superoxide dismutase (SOD) and catalase (CAT)), and non-enzymatic antioxidants (nicotinamide adenine dinucleotide) and reduced glutathione (GSH), and exogenous antioxidants, such as some vitamins and metals [67]. The SOD enzyme constitutes the first line of defense against free radicals by catalyzing the dismutation of O_2_^−^ to H_2_O_2_ and oxygen decreasing O_2_^−^ concentration, which damages the cells at an excessive concentration [68]. H_2_O_2_ formed is not a radical, but it is rapidly converted by the Fenton reaction into ^•^OH radical which is very reactive. For this reason, there are two antioxidant enzymes, catalase and the selenoprotein GPx, responsible for reducing it. GPx neutralizes H_2_O_2_ by taking hydrogens from two GSH molecules resulting in two H_2_O and one oxidized glutathione (GSSG). GR, an NADPH-dependent enzyme, is fundamental in this step since it regenerates GSH from GSSG. The GPx enzyme is more important against high H_2_O_2_ levels; however, catalase acts preferentially when there are low concentrations of H_2_O_2_. Besides, GPx is also responsible for detoxifying other lipid peroxides (LOOH) to the corresponding alcohol (LOH). The defense mechanism of endogenous antioxidant enzymes is shown in Figure 2. 

### 1.2. Se and MetS

#### 1.2.1. MetS Definition and Characteristics

MetS is characterized by the simultaneous presence of risk factors that contribute to cardiovascular disease (CVD) and diabetes [69]. These risk factors include elevating fasting glucose, high blood pressure, hypertriglyceridemia, low high-density lipoprotein cholesterol levels, and obesity (particularly abdominal adiposity). 

The first regularized definition of the MetS was proposed in 1998 by the World Health Organization [70], although this definition is not internationally accepted, being defined by various organizations (National Cholesterol Education Program-Adult Treatment Program III (ATP III), International Diabetes Federation). In 2009, a Joint Interim Societies (JIS) MetS definition was introduced, in order to alleviate discrepancies between previous guidelines [71]. They proposed common criteria for the clinical diagnosis of the MetS. The clinical criteria are elevated waist circumference (population- and country-specific definitions), hypertriglyceridemia (≥1.7 mmol/L), low HDL cholesterol level (<1.0 mmol/L in men and <1.3 mmol/L in women), high blood pressure (systolic blood pressure ≥130 mmHg, and/or diastolic blood pressure ≥85 mmHg), and elevated fasting glucose (≥100 mg/dL). According to this statement, for a diagnosis to be made, three of five of the proposed criteria should be met. This definition recognizes that the risk associated with a particular waist measurement will differ in different populations and that IR is also considered a linking factor to MetS since in most MetS patients abdominal obesity and IR appear [71].

The established relationships between MetS and T2DM and CVD are amply demonstrated, but the interest in MetS and visceral obesity is renewed, as it has also been related to other chronic diseases. In this context, it is shown that the most dangerous adiposity profile includes excessive amounts of both visceral adiposity and liver fat, which is the prevalent form of IR and MetS, being proposed as a marker of them [72]. Finally, in recent years, with precision lifestyle medicine, there has been a tendency to take into account the genetic profile of the individual as well as their environment and lifestyle [73]; these facts should also be taken into account in MetS patients.

#### 1.2.2. MetS and OS 

In clinical and experimental research, OS is commonly associated with MetS, since it contributes to obesity, diabetes, atherogenesis, hypertension, and other CVD. In most of these circumstances (even in MetS) chronic low-level inflammation appeared, and this event is deeply associated with a chronic whole-body OS generation [74]. Currently, new studies address the link between OS and MetS in the physiopathology of different biological systems, especially in mitochondria, since these organelles play a crucial role in cell physiology [75]. Mitochondria are implicated in metabolism, as they are involved in the production of energy (ATP) by the respiratory chain, in the metabolism of carbohydrates, amino acids, and lipids, and in apoptosis. The ROS produced in the mitochondria contribute to mitochondrial damage, which affects the cellular redox signaling, leading to a wide range of pathologies that comprise metabolic disorders [66]. Changes in ATP generation affect the activity of the energetic cellular sensor AMPK, modulating cellular energy expenditure.

OS is associated with MetS, but whether it is the cause or the consequence is not clear; however, many observations argue that both are true. For instance, during MetS SOD is down-regulated along with the selenoprotein GPx [76]. There is a strong correlation between NOX activity, together with low GSH deposits, and increased OS in MetS patients [77]. 

Since OS has emerged as a central player in chronic metabolic diseases such as MetS, multiple relevant markers need to be identified to clarify the role of ROS in the etiology of MetS. In this context, there are several clinical trials related to the use of the antioxidant Se as a useful marker and/or therapeutic approach to counteract OS and alleviate MetS symptoms. Moreover, Se, when connected to free radicals, can block the nuclear transcription factor kappa-B (NF-kB), leading to an inflammation reduction, as well as a reduction of the vascular cell adhesion molecule [78,79]; both circumstances usually appear during MetS instauration [80]. 

#### 1.2.3. Se and MetS in Human

Se is involved in various metabolic processes; however, there is limited and controversial research related to Se status and/or supplementation and MetS in humans. Since, as it was previously described, both high and low Se statuses are related to metabolic disorders.

Yuan et al. in a Chinese population found a dose-response relationship between plasma Se and risk of MetS, reporting higher plasma Se levels and major risk of MetS and hyperglycemia, though this association was significant only in women [81,82], according to the results of the IMMIDIET studies. Previously, in the Lebanese adult population, a positive correlation between plasma Se levels and the components of the MetS was also found [83]. However, Ford et al. did not find a significant difference in Se levels among American patients with or without MetS [84]. Similar conclusions were found by Tajaddini et al. in a clinical systematic review, probably due to differences in the study design and the population observed among researchers [85]. In 2017, Gharipour et al. demonstrated a significant decrease in circulating SelP levels according to MetS status in Iranian patients with documented CVD [86]. The same authors, in a systematic review, concluded that SelP is the best indicator for Se nutritional levels; that high levels of Se may increase the risk of MetS, while the lack of it may also promote MetS, and that for Se human supplementation, the selenium-yeast form is the best one [87]. However, this supplementation should not be recommended for cardio-metabolic risk prevention in populations with adequate Se status. In 2019, they also found that there were no differences in SelP genotypes between MetS and non-MetS subjects [88]. Studies in Brazilian adolescents between 12 and 17 years old found that the prevalence of the MetS in this population was 2.6% and that there was no association between the MetS and Se intake. Nonetheless, this lack of association was attributed to the adequate Se intake in 100% of the studied population [89].

Relative to the use of Se supplementation in MetS patients, Tabrizi et al., in a meta-analysis of randomized controlled trials, analyzed the effect of Se administration on glucose metabolism and lipid profiles in MetS patients [90]. This review described that Se supplementation may lead to an improvement in serum insulin levels and in insulin sensitivity, but it does not affect HOMA-IR and lipid profiles. Gharipour et al. found that Se supplementation did not affect plasma Se levels but slightly increased SelP serum level after a 2-month intervention in MetS patients [91]. This trial suggests that further studies should investigate the long-term use of Se supplementation in MetS patients. Recently, Retondario et al., in a systematic review, studied the association between Se intake and MetS, finding no association between Se intake and MetS in three studies; an inverse association in three others, and a direct association only in one [80]. They concluded that Se intake and MetS are not clearly associated in adults and the elderly. 

Therefore, in humans, due to the great variability of factors such as previous Se status, gender, the population under study, diet, lifestyle, smoking conditions, or the cardiovascular treatment, to name but a few; Se supplementation should still be limited for MetS treatment. However, since the sample analyzed in humans is mainly plasma; and the plasma level of metabolites is not a simple reflection of changes in tissue levels of the same metabolites [92], Se and selenoproteins’ tissue levels should be longer analyzed in experimental animal models. 

These experimental tissue studies are gaining greater importance since the prevalence of MetS is increasing worldwide and not only in adults, the pediatric age group is greatly affected as well [93]. Another important group is pregnant women since during a healthy pregnancy maternal organs and placenta are challenged to adapt to the increasingly physiological changes related to energy and metabolism. MetS appears in 25% of pregnant women and it has been outlined as one of the reasons related to MetS increase in pediatric age through the metabolic programming theory [94]. 

## 2. Metabolic Programming and Se

### 2.1. Se Implications in Metabolic Programming

The theory of the origin of health and disease (the developmental origins of health and disease) proposes that the homeostatic system affected during gestational and early postnatal development impedes the ability to regulate body weight after birth, particularly with regard to high energy intake, resulting in adult obesity and metabolic diseases [95]. These stimuli lead to a mismatch between prenatal and/or neonatal metabolic programming, which induces an increased risk of disease in adulthood [96]. According to that, fetal programming occurs when the optimal environment in which the fetus grows is disrupted by insults during prenatal development, inducing changes in the metabolic state and the susceptibility of adults to develop several chronic diseases such as metabolic dysfunctions and CVD [97,98]. Therefore, fetal programming is a well-known term that relates intrauterine nutrition to the development of diseases in adult life. Early postnatal programming term includes the postnatal period where the newborn presents a very active and rapid growth, which is also influenced by environmental factors, such as the breastfeeding period. The term suggests that nutritional changes during this neonatal period lead to a greater risk of disease later in life.

OS is one of the fundamental insults related to adverse fetal programming outcomes, leading to intrauterine growth retardation (IUGR) and abnormal tissue development, affecting gestational parameters, as well as endocrine metabolic balance and pregnancy disorders [99]. The embryo is highly susceptible to oxidative damage since the environment that surrounds it is poor in oxygen and has low antioxidant capacity. In addition, in the placenta there are numerous changes at the oxidative level, such as an increase in NOX and antioxidants, affecting the exchange of oxygen between mother and fetus and thus producing abnormalities due to hypoxia [100]. During the early postnatal programming (breastfeeding period), OS also plays an important role in mothers and neonates, since it compromises an appropriate lactation process which leads to growth retardation [36,101,102,103]. In this context, studies in humans and animals have revealed that the antioxidant Se is essential for maternal health and offspring development during reproductive periods. 

### 2.2. Studies in Humans

Rayman et al. evaluated in pregnant women from the United Kingdom whether Se status before pregnancy or Se supplementation influenced the risk of developing pre-eclampsia and hypertension [104]. They measured blood and toenail Se concentration, plasma SelP concentration, and GPx activity; finding that women are at higher risk of pre-eclampsia and hypertension when they have lower Se levels, and that supplemented women presented higher levels of Se together with lower risk of gestational pathologies. Grieger et al., in Australian pregnant women, demonstrated that those who have low plasmatic Se levels have a longer time until pregnancy and a higher rate of subfertility [105]. Hofstee et al. investigated in Australian women the relationship between maternal Se serum levels and thyroid hormones (THs) status during pregnancy since thyroid disorders are one of the most common endocrine disorders affecting women commencing pregnancy [106]. They found a decrease in free T3 levels, an increase in thyroid peroxidase antibodies, and a major incidence of gestational diabetes mellitus (GDM) in the low serum Se cohort. These alterations can lead to undesirable pregnancy outcomes.

Barman et al. studied the association between gestational length and preterm delivery (PTD) in 72.025 Norwegian women, and maternal Se intake and status [107]. An increase in gestational duration and a reduction in PTD were found associated with a higher Se intake during gestation, but not after Se supplementation, probably due to the fact that about half of the women had an optimal dietary Se intake. Monnagi et al. (2021), in samples collected from different populations (17 international birth cohorts with diverse ethnic backgrounds and geographic distributions), analyzed the relationship among maternal Se concentrations during pregnancy with PTD risk and gestational duration [108]. In this study, the hypothesized mechanisms to link Se and PTD risk were attributed to the selenoproteins GPx3, SelP, and TXNRD, for their antioxidant and anti-inflammatory action. However, they concluded that Se supplements cannot be considered as a general strategy to prevent PTD or increase gestational duration, since it depends on maternal Se status. Other studies analyzing prenatal Se exposures by measuring its concentration in maternal blood samples, demonstrated in male infants a positive association between Se and the birth weight, and in female infants to the index weight/height [109]. They concluded that prenatal exposure to Se is related to birth outcomes and that infant sex may modify these associations. Modzelewska et al. studied the relation between maternal Se intake and Se status during gestation and PTD and IUGR [110]; finding that mothers with a Se intake <30 μg/day lead to an increased risk of neonatal death. However, those with Se intakes >120 μg/day did not show this mortal association. 

In conclusion, it is clear that low Se levels during gestation are related to maternal and neonate diseases such as pre-eclampsia, hypertension, subfertility, GDM, PTD, IUGR, and neonatal death, since, among others, it is necessary to maintain an adequate oxidative balance [111]. However, the Se supplementation in these cases is not easy to apply, since it depends on maternal Se status, Se dose and source, and infant sex. Once more, because the human sample used in these studies is serum, more animal research is necessary in order to obtain tissue fetal and neonate information.

### 2.3. Studies in Animals

Numerous animal studies in different species from research and agriculture have evidenced a key Se role on fetal and early programming, showing that it is involved in maternal endocrine regulation, placental development as well as in fetal, and neonatal growth and development [39,111,112,113,114,115,116]. 

Like in humans, animal Se-deficient studies during reproductive periods show that low dietary maternal Se intake is related to IUGR and miscarriages. However, in animal models, the repercussions of Se supplementation during gestation and lactation periods have been extensively studied. In general terms, elevated dietary maternal Se supply from an organic or an inorganic form improved birth and weaning weight in animals, such as sheep, pigs, and rodents [113,117,118,119]. Despite the fact that supra-Se supplementation can have deleterious metabolic effects since this element induce IR and diabetes in mice, rats, and pigs [114], in agriculture, maternal supplementation is broadly used in broilers, calves, pigs, sheep, and goats in order to increased progeny development [113,117,118]. 

Animal models are also very interesting since they give information related to maternal Se status and cardio-metabolic programming processes which appear later in life. For instance, Hofstee et al. found that maternal Se deficiency in rodents induces growth restriction, with significant decreases in fetal heart and kidney size, which predispose offspring to cardiovascular and renal dysfunction in later life [111]. These authors concluded that cardiovascular alterations took place when IUGR appear and that they are probably related to the impaired thyroid dysfunction that Se deficiency offspring present by decreasing the activity of the selenoproteins DIOs [120,121]. Laureano-Melo et al. observed that maternal Se supplementation to Wistar rats was able to program carbohydrate and lipid metabolism, endocrine homeostasis, and feeding behavior, even in the adult offspring [122]. They suggested that metabolic effects were induced by THs disbalance together with the antioxidant mechanisms. 

Therefore, Se homeostasis is important for mother and progeny, not only for its effects modulating the oxidative balance, but also for its involvement in protein synthesis, regulation of the cell cycle, remodeling tissues, and modulation of metabolism–endocrine-energy balance [32,112,123,124]. Se deficiency altered the placental nutrient transporter expression, decreasing fetal glucose concentration, which contributes to the so-called growth restriction [112]. Selenoproteins (such as GPx1, SelP, and DIOs) are necessary for the correct synthesis and function of insulin, insulin-like growth factor-1 (IGF-1), and thyroid hormones (THs); all of them being hormones implicated in the correct fetal and neonatal growth [36]. GPx2, which is mainly expressed in the GIT system, protects intestinal cells from OS and inflammation improving their structure and function; increasing nutrient absorption, and promoting body growth and development [125,126,127]. Additionally, selenoproteins are also involved in the central endocrine regulation of appetite and energy homeostasis, affecting growth and development [128]. Finally, GPx4 avoids oxidation of mitochondrial cardiolipin, which in turn facilitates the cytochrome-c release and activates the apoptotic signaling cascade [129], as it is also related to NF-kB activation and inflammation [130]. When this protein is suppressed, such as in GPx4 knockout mice, embryos are non-viable, dying by gestational day E 8.5 [131].

**Figure 3:**
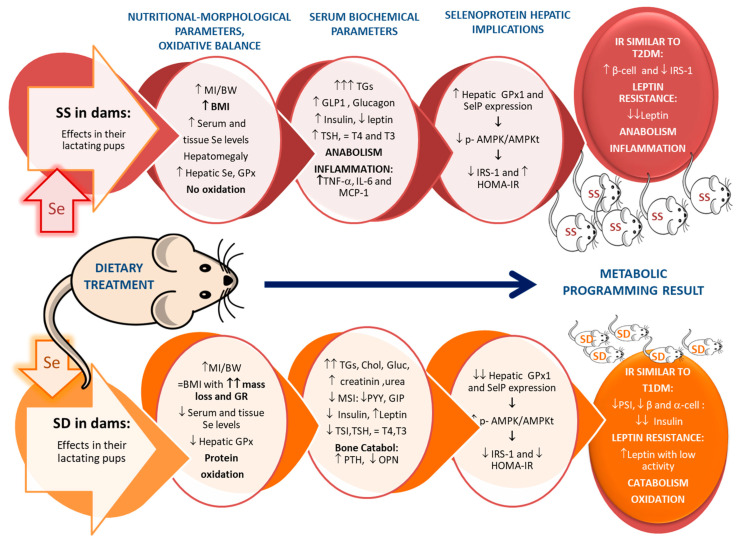
Maternal selenium status and metabolic programming. Effects of maternal Se diet on their offspring: nutritional-morphological parameters and oxidative balance; biochemical parameters; hepatic selenoproteins’ expression and metabolic programming results. SS: selenium supplemented dams (0.5 ppm); SD: selenium deficient dams (0.01 ppm). MI: milk intake, BW: body weight; BMI: body mass index, GR: growth retardation, GPx: glutathione peroxidase, TGs: triglycerides, GLP1: glucagon-like peptide-1, TSH: thyroid-stimulating hormone, Chol: cholesterol, Gluc: glucose, MSI: intestinal mucosa somatic index, PYY: peptide YY, GIP: gastric inhibitory polypeptide, TSI: thyroid somatic index, PTH: parathyroid hormone, OPN: osteopontin, SelP: selenoprotein P, AMPK: AMP-activated protein kinase, IRS-1: insulin receptor substrate 1, HOMA-IR: homeostatic model assessment for insulin resistance, IR: insulin resistance, T2DM: type 2 diabetes mellitus, T1DM: type 1 diabetes mellitus, PSI: pancreas somatic index, ↑: increase, ↓: decrease.

Animal experimental protocols have used different animal species, different dietary Se forms (organic or inorganic) with different doses, and most of them are developed only in Se-deficient pups or Se-supplemented ones. Therefore, to obtain simultaneous information concerning how the Se intake by dams is involved in programming endocrine energy balance in the progeny and its homeostasis, three experimental groups of dam rats were used in our research laboratory. The groups used were control (Se: 0.1 ppm), Se-supplemented (SS) (Se: 0.5 ppm), and Se-deficient (SD) (Se: 0.01 ppm); Se was supplied as sodium selenite to dams. After birth and at the end of lactation (21 d old), different parameters were analyzed in the offspring. We demonstrated that maternal Se status (high- or low-Se diets) is profoundly involved in metabolic programming by modulating IR, OS, and energy homeostasis [32,124]. In both situations, insulin, leptin, and HTs signals are disrupted and, therefore, the long-term endocrine signal for energy balance regulation is profoundly disturbed, but with different repercussions: high-Se diet leads to an anabolic profile, while a low-Se diet is related to an extreme catabolic energy imbalance. In Figure 3 all these changes can be observed, divided into nutritional-morphological parameters and oxidative balance; serum biochemical parameters; selenoprotein hepatic implications, and metabolic programming results.

Therefore, in terms of metabolic programming results, maternal Se supplementation leads to high insulin non-operative secretion resulting in obesity, anabolism, inflammation, and low leptin levels, all parameters similar to those found in a T2DM process in pups. However, SD pups present a metabolic profile similar to that of pups with type 1 diabetes mellitus (T1DM), with extremely low insulin secretion, leading to severe growth retardation, a catabolic status, underdeveloped endocrine glands, oxidation, low gastrointestinal-anorexigenic signals, and high non-operative serum leptin levels. These results point to Se as an important element implicated in metabolic programming, which could modulate metabolic disorders such as IR, IUGR, or MetS.

## 3. Selenium, MetS Programming

MetS (a syndrome deeply related to OS) can appear during gestation and lactation in dams, leading to OS and energetic and metabolic changes in their offspring that, according to the fetal programming theory, will produce cardiovascular and metabolic diseases as IR and diabetes later in life [132]. This programming phenomenon has been described in the literature in clinical trials and animal research [133,134,135]. Since it has been demonstrated that maternal dietary Se is intimately implicated in metabolic programming by modulating IR, OS, and energy homeostasis [32,124], we thought that maternal Se status could play an important role in this pathology during gestation and lactation in mothers and in the offspring. Therefore, we initiated a research line with the aim of elucidating Se’s implication in MetS programming in rats, in order to gather information in all the tissues implicated. These studies will provide important information to valorize the use of maternal Se diet as a therapy in MetS-reprogramming.

### 3.1. High Fructose Diet as MetS-Programming Model

**Figure 4:**
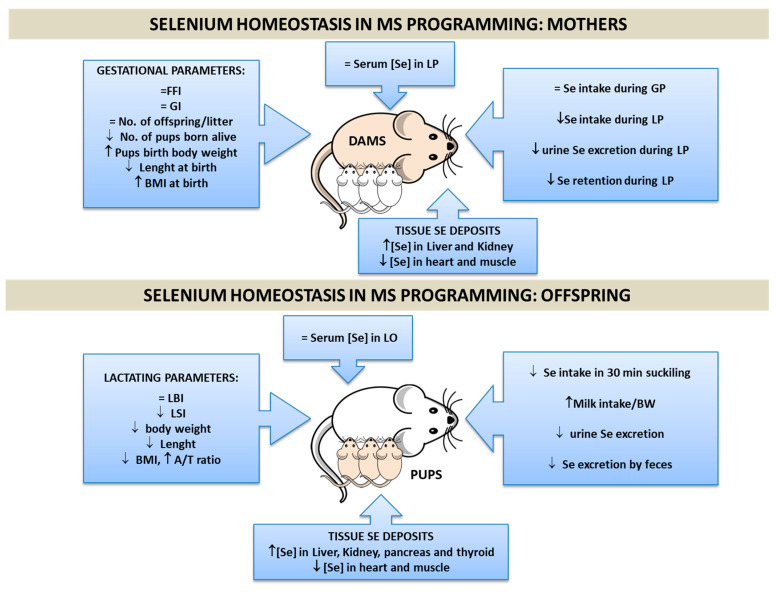
Selenium homeostasis in MetS programming: results in dams and in their offspring. Se intake, excretion, serum concentration, and tissue deposits were analyzed in dams (during gestation process: GP and lactation process: LP) and in their breastfeeding pups (lactating offspring: LO); together with gestational parameters (FFI: female fertility index (nº of pregnancies/nº of mating) × 100, GI: gestational index (nº of successful births/nº of pregnancy rats) × 100) and lactating parameters (LBI: live-born index (nº of pups born alive/nº of pups born) × 10, LSI: lactation survival index (nº of total offspring/nº of dead offspring/nº of total offspring) × 100, and A/T ratio: (abdominal circumference/thoracic circumference) × 100), ↑: increase, ↓: decrease.

There are different animal experimental models to induce MetS in progeny, such as a maternal low-protein diet, or a high-fat diet [135]; but, recently, a high fructose intake is the most commonly used [136]. This research group used a well-established experimental-Met model based on maternal high fructose diet (HFruD) (65%), that induces MetS in rats in 3 weeks [137]. We administered fructose (65%) in solid diet (with control Se content) to female rats during the induction (3 weeks), preconception (1 week), gestation (3 weeks), and lactation (3 weeks) periods [138]. This model in dams at the end of lactation leads to changes in the BMI, joint to a bad fat profile, hepatomegaly, hypertriglyceridemia, unaltered serum Gluc values, decreased insulin serum levels, increased systolic and mean BP, decreased heart rate (HR), microalbuminuria, hypernatremia, and hyperaldosteronemia [139]. All these complications confirm that dams suffer MS, which affected gestational success since they presented a lower number of pups born alive (Figure 4) [138]. These maternal disturbances influenced the metabolism of their offspring. HFruD pups had higher BMIs at birth, but, like their mothers, it was lower at the end of lactation. They also present low insulin levels and β-cell function, joint to normal serum Gluc, and increased TGs levels [124]. Maternal HFruD alters the development of lactating pups differently according to sex [140]. Female pups present hepatomegaly and extremely low insulin serum levels, indicating a greater metabolic disruption in these pups; in male pups, growth and development are affected to a greater degree. Both sexes presented albuminuria, hypernatremia, and hyperaldosteronemia, leading to a hypertensive status [141,142]. All these changes difficult the weaning process as it is expressed in Figure 4. Consequently, maternal HFruD exposition during the reproductive states leads to MetS early programming in their offspring, in part by generating OS [143]. In this context, Se implication in MetS programming is a difficult issue, since its beneficial action avoiding OS could be counteracted by its implication in the IR process. In order to deepen into the knowledge of the possible role that Se plays during gestation and lactation in this pathology, and its transmission to the progeny, our group studied for the first time Se body distribution in dams, which develop MetS during gestation and lactation and in their pups (Figure 4). 

### 3.2. Se Homeostasis in MetS Programming

MetS dams have normal Se serum values during the whole experimental process, despite the fact that during lactation they ingested a lower amount of food and therefore of Se [138]. They excreted less Se by urine, probably trying to maintain normal serum and milk Se levels. This effort was insufficient, and Se retention decreased in MetS dams, as it was found when the apparent Se balance was measured. Se deposits in the liver and kidneys were elevated in MetS dams, whereas in the heart and muscle there was a significant depletion. This shows that Se distribution during MetS in lactating dams is controversial. It is well established that HFruD produces OS through ROS formation, especially in the liver and kidneys [143,144]. Thus, the Se increase in these tissues could enhance GPx enzyme activity, protecting them from oxidation. However, this is a double-edged sword in MetS, since it has been demonstrated that overproduction of the selenoproteins GPx and SelP in the liver produces IR, Gluc intolerance, and dyslipidemia [46]. The depletion of Se found in the heart shows, for the first time, that heart Se deposits could play a primordial role in the myocardial dysfunction found in dams with MetS; since Se deficiency contributes to fibrosis and diastolic dysfunction development [145]. Skeletal muscle is one of the greatest Se-storing organs [146]; in MetS dams, its Se stores are being destroyed in order to increase Se levels in other tissues. This Se depletion could increase ROS production and contribute to disrupting the insulin signaling pathway since skeletal muscle is the largest insulin-sensitive tissue in the body [147].

Regarding Se homeostasis in offspring at the end of weaning, the pups from MetS dams received less Se via milk, but their serum Se levels were unaltered [140]. MetS-exposed pups excreted less Se via feces and urine, trying to retain this element. However, this effort did not work, because we found, for the first time, that tissue Se deposits were altered in MetS-exposed pups. Like happened in their dams, both genders of pups showed lower levels of Se in the heart and muscle, and higher levels in the kidney, pancreas, and thyroid. However, only female pups presented a significant repletion of Se in the liver, having the same pattern of Se distribution as their mothers. Accordingly, this shows that this behavior could be typical in females.

In conclusion, it has been shown that maternal MetS causes changes in Se tissue deposits of suckling pups. These changes will contribute to different tissues’ selenoprotein expression and to alterations in tissues’ oxidative balance and function, contributing to CVD, endocrine alterations, and IUGR. 

### 3.3. Selenoproteins’ Homeostasis in MetS Programming: Pathophysiological Repercussions

**Figure 5:**
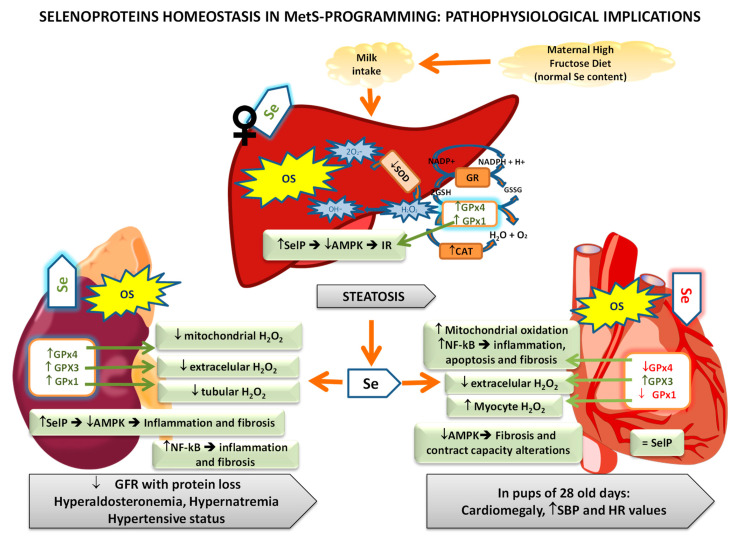
MetS programming and selenoprotein expression: pathophysiological implications. Liver, kidney, and heart Se deposits and selenoproteins (GPx1, GPx3, GPx4, and SelP) expression in breastfeeding MetS-exposed pups are affected in a different way, however, in the three tissues, OS was established. These selenoproteins’ imbalance is related to changes in ROS (H_2_O_2_) generation and OS, in nuclear factor kappa-B (NF-kB) expression and inflammation and fibrosis, and in AMP-activated protein kinase (AMPK) expression and energy cellular balance, in part related to pathophysiological alterations such as insulin resistance (IR) and liver steatosis, low glomerular filtration rate (GFR), hyperaldosteronemia, hypernatremia, high systolic blood pressure (SBP), cardiomegaly, and high heart rate. OS: oxidative stress, SOD: superoxide dismutase, CAT: catalase, GR: glutathione reductase, GPx: glutathione peroxidase, GSH: reduced glutathione, GSSG: oxidized glutathione, ↑: increase, ↓: decrease.

Numerous studies evidence the role of selenoproteins in different tissues (mainly liver, kidney, and heart) preserving the cell integrity from OS, representing a promising therapeutic tool in the treatment of metabolic diseases related to OS such as MetS [148,149]. Figure 5 summarizes the main selenoprotein changes observed in MetS-exposed pups after HFruD and their possible pathophysiological repercussions.

The liver is a key organ in the whole-body Se homeostasis since absorbed Se from the diet is transported to the liver, where it is metabolized to Sec and incorporated into selenoproteins, including SelP, the main source of Se to other tissues of the body [15]. With the MetS model previously described, it has been proved that OS takes place in the liver of MetS-exposed pups at the end of lactation, mainly increasing protein oxidation; however, the implication of Se in this process is different among sex [140]. Hepatic antioxidant balance was compromised both in female and male offspring since the antioxidant activity of SOD was decreased (Figure 5). Numerous authors have also found a decrease in SOD activity in adult rats and humans with MetS [150,151,152]. In this context, Liu et al. studied the association between SOD activity and MetS progression, observing that the activity of this enzyme showed a linear descending trend according to the sequential progression of the MetS different components, such as impaired insulin sensitivity and β-cell dysfunction [153]. 

With regard to GPx and CAT, only female pups showed a high hepatic activity of catalase and GPx; however, they also presented higher lipid oxidation [140]. This augment in the liver selenoprotein GPx activity of female pups is due to the higher hepatic Se deposits found in them, a fact that does not occur in males. Ojeda et al. proposed that hepatic Se repletion during MetS is a typical behavior of the female gender since it was also produced in their dams [140]. This Se repletion improves GPx activity, acting more efficiently against the OS provoked by MetS. However, this beneficial action could have a dual function in MetS, since GPx not only acts against oxidative damage, one of the main triggers of this pathology, but also it eliminates the “good” ROS necessary to initiate the insulin signaling pathway (Figure 1); this contributes to IR instauration. This effect is consistent with the hepatomegaly and steatosis only found in female pups. The so-called dual role of liver GPx was also observed in MetS dams [138]. 

According to the higher hepatic Se deposits and GPx activity found in females MetS-exposed pups, both GPx1 and SelP (selenoproteins related to IR induction) were highly expressed [54]. Both selenoproteins, as well as GPx4, are the most expressed selenoproteins in the liver [154]. Moreover, SelP (apart from its action decreasing AMPK activation) provides the Se necessary to the biosynthesis of GPx1, promoting its antioxidant activity and its possible action on the insulin signaling pathway. According to that, MetS-exposed female pups presented lower hepatic AMPK-p and IRS-1 levels, and extremely low insulin serum levels, confirming that the IR process is taking place. Since SelP inhibits AMPK activation, it also affects hepatic fatty acid homeostasis [155]. AMPK increases catabolic pathways and decreases anabolic pathways to maintain intracellular energy balance and regulate whole-body energy metabolism [156]. Thus, the inactivation of hepatic AMPK leads to an increase in fatty acid biosynthesis and steatosis; this last effect was found in female pups.

However, neither GPx1 nor SelP were increased in male MetS-exposed pups, consistent with a lower hepatic OS and a non-affected AMPK activity [54]. Nevertheless, they also presented low insulin serum levels and IRS-1 expression in the liver but these decreases were not as significant as in females. Therefore, more mechanisms are implicated in this process. According to that, both groups of animals present a significantly higher GPx4 expression in the liver. GPx4 is the only GPxs family member that specifically scavenges lipid hydroperoxides in membranes, including the mitochondrial one, where it plays an important antioxidant role [157]. This selenoenzyme is essential during early development, since mice lacking GPx4 die early in embryonic development, shortly after gastrulation (E7.5) [131]. Besides, it was recently demonstrated that GPx4 deficiency in obese mice causes marked OS, which leads to enhanced lipid peroxidation and carbonyl stress in the liver, exacerbating IR, steatosis, and heart dysfunction, all of which are risk factors to MetS [158,159]. Our results confirm that MetS-exposed pups make a clear effort to increase GPx4 activity, perhaps because it has protective effects against the OS that occur in conjunction with metabolic disorders, as defended by Katunga et al. [158]. Therefore, hepatic GPx4 plays an important role during the MetS programming instauration.

Vahter et al. reported that Se deposits in kidneys are age-dependent and that these levels increase during the postnatal period [160]. In the MetS-programming model proposed, pups presented Se kidney repletion independently of their sex; and high tubular Se reabsorption, since these pups have lower Se relative clearance [141]. Therefore, the expressions of the selenoproteins analyzed were all increased (GPx1, GPx3, GPx4, and SelP). GPx3 is mainly expressed in the kidney and have extracellular antioxidant activity, being very important its antioxidant activity in plasma [161,162,163]. The increase in GPx1 expression improved tubular antioxidant activity; however, lipid oxidation appears (Figure 5). This oxidation triggered changes in the plasma membrane composition of kidney tubular cells and interferes with the main carriers’ function, such as with Na^+^K^+^-ATPase [164,165], consequently increasing Na^+^ reabsorption and K+ excretion and generating hypernatremia in MetS pups. GPx4 plays a vital role in reducing the hydroperoxide group of fatty acids in the mitochondrial membrane [157], avoiding cytochrome-c release and so, apoptosis. Moreover, GPx4 is related to transcriptional factor NF-kB, intimately linked to inflammation, and inversely related to apoptosis [166,167]. MetS-exposed pups present high NF-kB expression in their kidneys. This transcriptional factor also contributes to fibrosis by activating different signaling pathways [168]. The increased expression of SelP in the kidney of MetS pups is one of the causes of the AMPK inactivation found. This energetic sensor, in the kidney, protects against inflammation and fibrosis in models of diabetic nephropathy [80]; therefore, this situation is contributing to inflammation and fibrosis generation.

All these biochemical alterations lead to several kidney functional alterations, such as low glomerular filtration rate, low urinary flow, high albuminuria, greater water tubular reabsorption, and high volemia. These pups also present hyperaldosteronemia, together with an altered function of Na^+^/K^+^-ATPasa provoked by OS, which produces hypernatremia. All these events contribute to a hypertensive status in these pups, as was demonstrated one week later [141]. Similar results have also been observed in children with low birth weight [169]. These kidney programming alterations are related in part to OS; therefore, Se and selenoproteins renal up-regulation could be beneficial since it avoids higher lipid oxidation. However, in order to analyze the possible global beneficial role of Se in kidneys during MetS exposure, more data are necessary to document the relationships between GPx4 and NF-kB, and SelP and AMPK in kidneys.

Se and selenoproteins play an important role in heart development, function, and cardioprotection [148]. In the HFruD-model used, heart Se deposits in pups were depleted without difference among sex, leading to lower antioxidant GPx1 and GPx4 expression and activity in cardiomyocytes [142]. Therefore, lipid and protein oxidation take place in cardiomyocytes, affecting correct heart function. In this tissue, GPxs decreased their activities and so, they could not cope with the high amount of ROS generated (Figure 5). The lower GPx1 expression found was directly related to cytoplasmatic oxidation in cardiomyocytes; and the lower GPx4 one to oxidative damage in the membrane of cells and organelles such as the mitochondrial, promoting apoptosis. GPx4 also modulated NF-kB expression [170], which was increased in MetS pups, leading to inflammation and fibrosis in this organ. 

GPx3 expression was not in consonance with heart Se deposits, being the only selenoprotein augmented in the heart. This might be because GPx3 comes directly from the plasma delivered by the kidney, where it was increased [141]. Therefore, GPx3, the only GPx with extracellular antioxidant activity, seems to be sequestered toward the heart matrix of MetS pups in an attempt to maintain its antioxidant balance, which was profoundly altered [142]. Moreover, GPx3 has a preventive role in CVD, since it avoids myocardial damage by decreasing Ca^2+^-dependent endoplasmatic reticulum stress (ERS), maintains the bioavailability of NO in the vascular system, and regulates the cardiopathology that accompanies diabetes [142,171,172]. In this sense, it was observed that GPx3 expression is reversed to insulin serum levels [171]. Additionally, it has also been found that cardiac hypertrophy may induce the expression of GPx3 in the heart to reduce H_2_O_2_ in the extracellular matrix [173]. The MetS-exposed pups presented low serum insulin levels, high GPx3 expression, and cardiomegaly.

Heart SelP expression was unaltered in MetS pups; however, no relationship among SelP and AMPK activation was found, since in these pups AMPK activation was decreased [142]. Low AMPK activity leads to a lower ATP generation, compromising the functionality and ability of the heart to contract, a tissue with a highly energetic demand. Moreover, in this organ, low AMPK activity leads to mTOR activation, protein synthesis, cytoskeletal network expansion, inhibited autophagy, and the appearance of cardiac fibrosis and hypertrophy [174,175], coinciding to the cardiomegaly found in MetS pups.

These molecular events provoked in MetS-exposed pups, in part by the decrease in selenoproteins’ expression, a modest increase in systolic blood pressure (SBP), and a high HR value one week after the end of the lactation period. This high HR seems to be due to a myocardial contractibility problem [142]. Therefore, MetS-exposed pups present important changes in the cardiac programming process, leading to myocardial fibrosis and hypertrophy, OS, inflammation, and heart contractibility problems. 

In the three tissues studied OS appeared, especially in the heart, finding antioxidant endogenous enzymes disruptions, some of them related to lower GPxs activities, like in the heart. It is clear that general OS is taking place in MetS-exposed pups, together with inflammation and alterations in cellular energy balance. All these events are related to the studied selenoproteins, since they have different antioxidant repercussions, by decreasing ROS in the cytosol, in the membrane of organelles, and/or extracellular fluids; contributing to modulate inflammation (NF-kB), apoptosis, and energy balance (AMPK). Despite the fact that they are increased or decreased in tissues, OS is still taking place; therefore, their antioxidant activities are insufficient, and Se supplementation seems to be a therapeutic approach to MetS-exposed pups. Nevertheless, is important to take into account the dual role of Se in hepatic IR genesis and renal inflammation and fibrosis via SelP. 

## 4. Selenium and Endocrine Energy Balance in MS-Exposed Pups

Apart from selenoproteins changes in their expression in tissues of suckling pups exposed to MetS related to IR, steatosis, and cardiovascular-renal dysfunction, antioxidant selenoproteins have been directly implicated in the central endocrine regulation of appetite and energy homeostasis by affecting the hypothalamus function [176]. Indeed, programming of appetite and energy expenditure may stem from the remodeling of hypothalamic structures and from an altered response to anorexigenic peptides [177]. Rats exposed to MetS during the intrauterine period, exhibit a greater degree of obesity if given high caloric food, indicating augmented energy conservation and altered feeding behavior [178].

### 4.1. Hypothalamus 

Specifically, in the hypothalamic arcuate nucleus (ARC), there are two cell populations related to food intake and energy expenditure: anorexigenic neurons, which decrease appetite and increase energy expenditure, express proopiomelanocortin (POMC)-derived peptides, such as α-melanocyte-stimulating hormone (α-MSH); they are known as POMC/CART neurons, and orexigenic neurons, which increase appetite and decrease energy expenditure, co-express neuropeptide Y (NPY) and agouti-related peptide (AgRP); they are known as NPY/AgRP neurons [179]. It has been observed that ROS and ERS significantly affect these hypothalamic neuronal populations that regulate global energy metabolism [180].

In the last five years, a prominent role for selenoproteins in the ARC function has been supported. Different selenoproteins are abundantly expressed in AgRP and POMC neurons, and their expression levels are regulated by nutrient availability [181]. High transcript levels of *Gpx1*, *Gpx3*, *Selenof*, *Selenok*, *Selenom*, *Selenot*, and *Selenow* have been reported. Despite the fact that not all of their functions are known, GPxs modulate ROS and ERS production in ARC, preventing the latter’s malfunction [176]. Schriever et al. demonstrated that GPx4 plays a physiological role in balancing metabolic control signals and inflammation in AgRP, but not in POMC neurons [182]. SelM has been described as an ER-resident oxidoreductase implicated in leptin signaling and energy metabolism by increasing TXNRD antioxidant activity in the hypothalamus [183,184,185]. Kremer et al. have found that the Se recycling enzyme selenocysteine lyase (Scly), which is necessary for selenoproteins’ synthesis, is implicated in the susceptibility of developing a MetS and it is decreased in the hypothalamus [186]. Moreover, Torres et al., using Scly-Agrp knockout mice, found a reduction in weight gain and adiposity [187].

Since ARC is located near the median eminence (ME), an area thought to lack the blood–brain barrier (BBB), it has a pivotal position to monitor circulating levels of hormones and nutrients, receiving direct peripheral information of peptides from blood. Peripheral endocrine signals are divided into long-term endocrine energy balance signals, like insulin and leptin and short-term energy balance signals, like peptides secreted from the GIT such as ghrelin (Ghrl), glucagon-like peptide-1 (GLP-1), glucagon-like insulinotropic peptide (GIP), cholecystokinin (CCK), oxyntomodulin (OXM), and peptide YY (PYY). Long-term signals give information about the energy stored since these peptides are secreted in proportion to the existing fat mass from the pancreas and adipose tissue. They directly stimulate POMC/CART neurons and inhibit NPY/AgRP neurons leading to anorexigenic effects. Moreover, these neurons send projections to other hypothalamic areas related to thyrotropin release hormone (TRH) secretion and to thyroidal endocrine regulation [188]. Short-term signals provide information about the food that is eaten and how much the stomach is distended. These peptides send information to the hindbrain and have anorexigenic effects via vagus, except ghrelin which directly inhibits POMC/CART neurons having orexigenic actions [189,190,191]. Therefore, ARC integrates the peripheral information and regulates appetite and energy expenditure by having orexigenic or anorexigenic functions [188]. 

**Figure 6:**
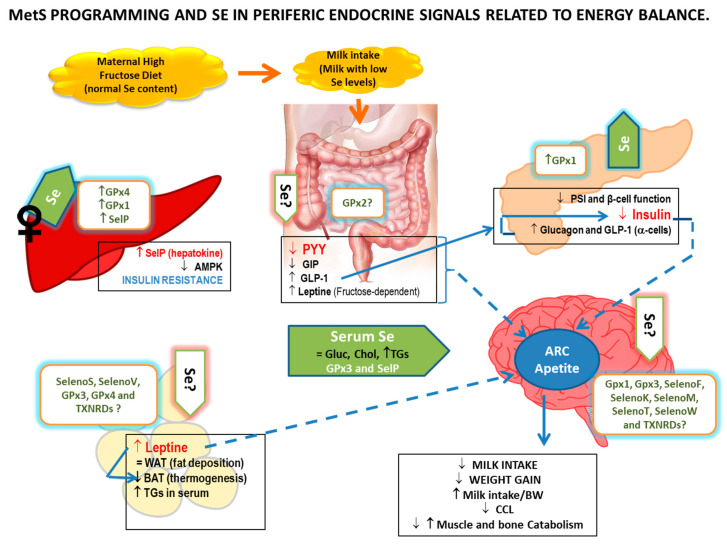
MetS programming and Se in peripheric endocrine signals related to energy balance. MetS exposition provokes changes in anorexigenics (PYY: peptide YY, leptin, and insulin) endocrine signals. Pups present lower milk intake, lower weight gain and length, and higher catabolism, pointing to the leptin resistance process. Hepatic IR process is taking place; however, in serum only triglycerides (TGs) levels are increased. In these processes, Se and selenoproteins (up- or down-regulated) could be implicated, since, in the gastrointestinal tract, pancreas, white adipose tissue (WAT), arcuate nucleus (ARC), and liver, Se and selenoproteins play an important antioxidant role. See text for more information. GIP: gastric inhibitory polypeptide, GLP-1: glucagon-like peptide-1, PSI: pancreas somatic index, BW: body weight, WAT: white adipose tissue, BAT: brown adipose tissue, CCL: cranium–caudal length, Gluc: glucose, Chol: cholesterol, ↑: increase, ↓: decrease.

To record information about how Se is involved in endocrine peripheral energy balance during MetS programming, peptides involved in short and long endocrine peripheral energy balance were analyzed together with pups’ appetite profile and Se status in the organs implicated in body energy metabolism like GIT, liver, pancreas, hypothalamus, and adipose tissue (AT) (Figure 6). With this information, we will get a simplified overview of functional aspects of Se-regulation of fatty acid, Gluc and protein metabolism, and energy homeostasis, through the interorgan crosstalk in MetS-exposed pups.

### 4.2. Peripheral Short-Term Signals

These energy signals are mainly formed by peptides secreted from the gastrointestinal tract and have digestive properties. HFruD affects GIT structure and function by different mechanisms, among them by generating OS in the small intestine and liver [192]. Enteroendocrine cells (EECs) distributed along the GIT release gut peptides in response to luminal stimuli (including nutrients and microbiome change), modifying the endocrine short-term energy balance signals, probably being affected by fructose exposition [193]. Kuhre et al. describe that fructose induces CCK, GLP-1, and PYY, but not GIP secretion in healthy young humans [194]. 

OS affects intestinal cell proliferation, differentiation, barrier function, and mucosal defenses [195]; the small intestine being one of the target organs of dietary Se [196]. The main antioxidant selenoprotein expressed in the epithelium of the GIT is GPx2, which also has anti-inflammatory properties and supports the growth of transformed intestinal cells [125]. It also affects EECs secretions, since a lack of GPx2 together with Se deficiency is accompanied by a decrease in GLP-1 and Ghrl [197]. A maternal Se-deficient diet affects GIT development and its endocrine function in their weaning pups [18], leading to a lower capacity to assimilate ingested nutrients and, therefore, retardation in the animals’ growth [31]. 

Despite the fact that Se deposits in the GIT of weaning MetS-exposed pups are not measured, we know that, as in Se-deficient breastfeeding rats, GIP and PYY are decreased in MetS-exposed pups [198]. Therefore, it is probable that GIT Se deposits are decreased and its integrity affected since these pups at the end of weaning also present a lower BMI and an important loss of mass and length. Apart from the growth impairment, MetS pups also present high serum TGs levels and low brown adipose tissue (BAT) depositions, consistent with the low PPY and GIP levels found. GIP together with GLP-1 are incretin gut hormones that increase the insulin glucose response [199]. Low GIP levels, contrary to the high GLP-1 serum values found in MetS pups, are in consonance with the low insulin serum values found in MetS pups. The higher levels of GLP-1 found in MetS pups are probably related to fructose intake since previous studies have determined that fructose significantly stimulates GLP-1 secretion in mice, rats, and humans without affecting GIP levels [194]. 

### 4.3. Peripheral Long-Term Signals: Pancreas and Adipose Tissue

Long-term signals provided by insulin from the pancreas and leptin from white adipose tissue (WAT), lead to anorexigenic effects and stimulate the secretion of hypothalamic TRH, increasing basal metabolism by stimulating thyroid gland hormones secretion [188]. In these tissues, Se plays a pivotal role, being an important nutrient in controlling food intake and energy expenditure. 

#### 4.3.1. Pancreas

MetS is a clustering of factors indicative of poor metabolic health; however, the main mechanism to explain its development is not clear. IR has most commonly been proposed for this role and is generally considered to be a root causative factor [200]. Since insulin is secreted by β-cells in the pancreas, this tissue plays a critical role in MetS. Pancreas development and its endocrine and exocrine functions are deeply related to pancreatic Se deposits. This mineral has well-documented anti-inflammatory, antioxidant and pro-apoptotic actions in this tissue [201]. Pancreatic β-cells, which are responsible for insulin synthesis, have an inherent deficiency in their capacity to cope with OS and they produce a large amount of ROS during insulin synthesis. In these cells, a correct GPx antioxidant activity has an important repercussion by avoiding pancreatic β-cells oxidation [202]. Moreover, the mass of β-cells declines with the progression of ROS [203]. Se restriction causes pancreatic atrophy with hypoinsulinemia by down-regulating selenoproteins and insulin signaling genes in the pancreas [202].

According to that and relative to metabolic programming, in previous studies, we have found that pups exposed to a low-Se diet have extremely low Se deposits in the pancreas, an underdeveloped pancreas, low levels of the insulin precursor C-peptide, and extremely low insulin secretion, which implies that β-cells are collapsed. Moreover, no increase in incretins (GLP-1 and GIP) and polypeptide P (PP) were found. This makes it evident that the insulin secretion process is almost null, and that not only are β-cell dysfunctional but also α-cells are dysfunctional, even when there is a deprivation of Se during gestation and lactation (Figure 3) [32].

In MetS pups exposed to HfruD, an increase in Se pancreatic deposits has been found; however, this effort was insufficient to ensure a correct pancreatic endocrine function [198]. These pups present an underdeveloped pancreas with a decrease in β-cell function and a drastically low insulin secretion, leading to a pancreatic profile similar to that of Se-deficient exposed pups and/or T1DM. However, the pancreatic endocrine profile in MetS pups was not so bad compared to that of Se-deficient pups, since in an attempt to increase insulin secretion, MetS pups have higher levels of the insulin secretion stimulators: glucagon and GLP-1. It was recently reported that GLP-1 is also produced from the same precursor as glucagon in pancreatic α-cells [204]. Therefore, the MetS model used in our laboratory does not seem to affect the exocrine pancreas and α-cells populations. The low insulin secretion found, joint to the resistance opposed to the hepatic insulin signaling mainly found in females MetS-exposed pups, complicate the correct function of this important hormone with growth function during breastfeeding in MetS-exposed pups (Figure 6). 

#### 4.3.2. Adipose Tissue 

AT is organized in a large organ divided morphologically and functionally into white (WAT) and brown adipose tissues (BAT). The first one stores lipids in form of TGs to allow intervals between meals, and the second one burns lipids for thermogenesis via high levels of the mitochondrial uncoupling protein 1 (UCP-1), combating hypothermia and obesity [205]. However, they present plasticity with the ability for reciprocal reversible transdifferentiation in response to physiologic needs by “whitening” or “browning” [206]. It functions as a critical regulator of energy metabolism regulating fat homeostasis. Great progress has been made in understanding the complexity of AT biology including inter- and intra-depot differences in adipocytes; its dynamic nature as a source of stem cells; the role of adipocytes from obese AT, which induces chronic low-grade inflammation, and its role as an endocrine organ that communicates with other tissues to regulate systemic metabolism through secretion of adipokines, such as the long-term appetite signal leptin secreted by WAT, inflammatory mediators, signaling lipids, and miRNAs packaged in exosomes. Therefore, altered AT function is intimately related to the pathogenesis of MetS [207]. In this context, HFruD promotes whitening of adipose tissue by decreasing UCP-1 expression in adult mice [208] and affects leptin secretion and function leading to leptin resistance (LR) [209,210,211,212]. This resistance could be due to intracellular deficits in cell signaling, a lower expression of leptin receptors, and/or impaired autophagy in WAT affecting its secretion [213,214]. Moreover, there appears to be a direct relationship between fructose and leptin, since long-term fructose consumption stimulates leptin production by the gastric mucosa and leptin increases intestinal GLUT-5 fructose transport activity [215,216,217]. Therefore, HFruD clearly affects long-term endocrine energy balance signals and body energy balance.

Different studies show that Se may modulate preadipocyte proliferation and adipogenic differentiation since it interferes with insulin signaling, regulates lipid accumulation and lipolysis, and also affects AT endocrine (including leptin), and immune functions [218]. This is due to the activity of different selenoproteins, such as the ER-resident selenoproteins (SelS, SelV, etc.) together with mitochondrial and cytosolic GPxs (mainly GPx3 and GPx4) and TXNRDs, which interfere with adipocyte development and functioning mainly by the modulation of redox homeostasis and ERS [182,219,220]. As it was mentioned before, these mechanisms are also implicated in the action of Se in ARC, actions which are related to hypothalamic leptin signaling and to central LR. Since leptin inhibits AgRP neurons promoting a negative energy balance, resistance to this anorexigenic action of leptin is strongly associated with obesity, IR, and MetS. A correct Se homeostasis by avoiding OS and ERS plays an important role in maintaining leptin sensitivity and a correct AgRP neuron response [186,187]. 

According to that, in Se-deficiency exposed pups, where all the tissues studied have extremely low Se deposits, AT mass was very poor [32]. However, these weaning pups have high serum leptin levels and probably LR, since they present an increase in relative milk intake (Figure 3). LR during neonatal state has been shown to increase both sympathetic activity and blood pressure in mice [221]. In a model of AgRP-Scly KO mouse, where hypothalamic selenoproteins’ expression is compromised since the protein Scly is necessary for a correct Se re-utilization, BAT had reduced lipid deposition and increased expression of the thermogenic marker UCP-1, showing that Se can influence energy homeostasis via AgRP neuron-mediated BAT activation [187]. If this situation is taking place in the model of Se-deficiency exposed pups explained before, the Se–AgRP–BAT axis could explain in part their extremely low weight and their increased catabolism.

Despite the fact that in the MetS model used by this laboratory, Se deposits in WAT and BAT do not have been measured in pups, we have described that WAT deposits were unaltered and BAT ones were significantly decreased, leading to an anabolic adipose ratio [222] (Figure 6). WAT deposits could be unaffected in MetS pups, probably because, like in adult animals, HFruD promotes whitening of adipose tissue by decreasing UCP-1 expression, collaborating to decrease BAT mass [208]. BAT plays an important role in linking poor fetal growth and the late development of adult metabolic diseases [223]. 

Despite having a normal amount of WAT, one of its main endocrine signals—leptin—is significantly higher in MetS pups. MetS pups, however, appear to develop peripheral LR since these pups have hepatic steatosis, low insulin secretion, low BAT deposits, and underdeveloped muscle and bone mass [54], all processes that leptin prevents [224]. 

As it was mentioned, THs are not only vital to the fetal nervous system development, linear growth, energetic metabolism, and thermogenesis, but also to the fluid balance and cardiovascular system [225], and the THs and MetS interrelationship has been studied [226]. Se is necessary for TH synthesis since forms part of the iodothyronine deiodinases (DIO1, DIO2, and DIO3), being the thyroid the organ with the highest Se content per gram of tissue [227]. In these MetS-exposed pups, thyroid mass was decreased, and Se deposits increased; however, TSH and free and total THs were significantly decreased [142]. Therefore, Se is not acting efficiently in these tissues, or at least not enough. The low HTs serum values are not in agreement with the catabolic process that is taking place in muscle and bone. Moreover, in MetS-exposed pups, THs are not controlling the secretion of TRH and TSH by negative feedback to maintain physiological levels of the main hormones of the HPT axis. Therefore, insulin, leptin, and TSH signals are disrupted in MetS-exposed pups, being the long-term endocrine signal for energy balance profoundly altered. Curiously, in these endocrine tissues (pancreas and thyroids), Se levels are up-regulated but are not acting properly or enough to maintain a correct secretive function. 

It could be concluded from this section that not only selenoproteins’ synthesis in the AT, pancreas, and GIT are necessary for normal sensitivity to leptin, insulin, and THs signals, but selenoproteins’ synthesis in the ARC is also necessary [228].

## 5. Conclusions

In conclusion, from a clinical point of view it is clear that the low Se levels during gestation are related to maternal and neonate metabolic diseases such as pre-eclampsia, GDM, IUGR, and probably MetS, since, among others, it is necessary to maintain an adequate oxidative balance. However, Se supplementation in these cases is not easy to apply, since it depends on maternal Se status, and Se dose and source. Moreover, since the human sample used in these studies is serum, animal research is necessary in order to obtain tissue fetal and neonate information. Experimental studies have shown that Se and selenoprotein homeostasis are unbalanced during MetS programming and are probably related to changes in oxidative balance, inflammation, energy balance, growth, renal and cardiac function, and endocrine regulation of metabolism. Se deposits and antioxidant selenoproteins (GPx) are up- or down-regulated in different tissues of MetS pups. However, OS appeared in all of them, their antioxidant activities being insufficient. Therefore, Se supplementation seems to be a therapeutic approach to avoid OS in MetS-exposed pups. However, taking into account the dual role of Se in hepatic IR genesis and renal inflammation and fibrosis via SelP, this decision is not so clear. Consequently, it is important to compare the general energy balance in MetS pups with those exposed to high or low Se supply in order to decide on Se supplementation or not. When dams received a low dietary Se supply, their pups suffered a metabolic alteration similar to T1DM with null insulin secretion and LR, OS, severe growth retardation, and an energy-wasting process. MetS offspring present a profile that is more similar to that of Se-deficient pups but is not so marked. Controlled Se supplementation should, therefore, be taken in mind if during gestation and/or lactation there are suspicions of endocrine energy balance dysfunctions related to OS, such as MetS. It seems that this therapy could be especially beneficial to induce heart reprogramming. However, more experimental research which examines Se as a potential reprogramming strategy during MetS must be taken into account before designing further clinical studies.

## Figures and Tables

**Table 1:** Main selenoproteins, their physiological functions, relation to Se status, and pathophysiological implications (modified from Qazi et al. [39] and Hariharan et al. [2]).

Selenoproteins	Physiological Functions	Relation to Se Status and Pathophysiological Implications
GPx1 (cytoplasmatic)	Antioxidant	Sensitive to Se intake. Cardiovascular diseases. Related to the endocrine system, intracellular signaling, appetite, growth, energy homeostasis, and IR.
GPx2 (gastrointestinal)	Antioxidant	Resistant to Se modifications. Intestinal cancer.
GPx3 (plasmatic)	Extracellular fluid antioxidant	Sensitive to Se intake. Cardiovascular protection.
GPx4 (membranes)	Membrane antioxidant. In sperm is a structural protein. Apoptosis	Resistant to Se modifications. Immune disorders, HIV, implications in male fertility, and mitochondrial function.
GPx6 (olfactory)	Homolog to GPx3	The knowledge of this GPx is very limited.
DIO1	Conversion of T4 to T3	Implications in immune thyroid disease and thyroid dysfunctions.
DIO2	Conversion of T4 to T3	Stable expression under low Se levels. Implications in immune thyroid disease and thyroid dysfunctions.
DIO3	Conversion of T4 to reverseT3	Implications in immune thyroid disease and thyroid dysfunctions.
TXNRD1	Antioxidant, redox regulation, cell signaling	Se dependent. In several types of cancer, there is an over-expression of TXNRD1.
TXNRD2	Antioxidant, redox regulation, cell signaling	Sensitive to Se intake.
TXNRD3	Antioxidant, redox regulation, cell signaling	Role in sperm maturation.
SelW	Antioxidant	Studies with tissue cultures of muscle and brain cells indicated that Se influenced SelW levels.
SelH	GSH synthesis	Implications in placenta oxidative stress.
SelV	Redox regulation processes	Although it is expressed in seminiferous tubules in mice, the exact role in spermatogenesis is unknown.
SelT	Endoplasmic reticulum homeostasis: promotes depletion of Ca stores and impaired hormone secretion	Unknow, although probably related with Endoplasmic Reticulum Stress.
SelP (plasma)	Main plasma Se transporter. Antioxidant. Indicator of Se status	Cancer, neurodegenerative diseases. Implicated in male fertility and maternal-fetal Se transfer. Apoptosis regulation. Related to the endocrine system, intracellular signaling, appetite, growth, energy, and IR.
